# Generating large field of view en-face projection images from intra-acquisition motion compensated volumetric optical coherence tomography data

**DOI:** 10.1364/BOE.404738

**Published:** 2020-11-04

**Authors:** Florian Schwarzhans, Sylvia Desissaire, Stefan Steiner, Michael Pircher, Christoph K. Hitzenberger, Hemma Resch, Clemens Vass, Georg Fischer

**Affiliations:** 1 Center for Medical Statistics, Informatics and Intelligent Systems, Medical University of Vienna, Vienna, 1090, Austria; 2 Center for Medical Physics and Biomedical Engineering, Medical University of Vienna, Vienna, 1090, Austria; 3 Department of Ophthalmology and Optometry, Medical University of Vienna, Vienna, 1090, Austria

## Abstract

A technique to generate large field of view projection maps of arbitrary optical coherence tomography (OCT) data is described. The technique is divided into two stages - an image acquisition stage that features a simple to use fast and robust retinal tracker to get motion free retinal OCT volume scans - and a stitching stage where OCT data from different retinal locations is first registered against a reference image using a custom pyramid-based approach and finally stitched together into one seamless large field of view (FOV) image. The method is applied to data recorded with a polarization sensitive OCT instrument in healthy subjects and glaucoma patients. The tracking and stitching accuracies are quantified, and finally, large FOV images of retinal nerve fiber layer retardation that contain the arcuate nerve fiber bundles from the optic nerve head to the raphe are demonstrated.

## 1. Introduction

Optical coherence tomography (OCT) is a contact-free in vivo imaging technique using near infrared light to create volumetric images of tissue in μm range accuracy [1]. It has long become of importance in the field of ophthalmology, being used for depth resolved visualization of the retina and cornea and has since become - next to fundus photography and Scanning Laser Ophthalmoscopy (SLO) - one of the key tools for diagnosing and progress monitoring of many eye-diseases [2,3]. While fundus photography and SLO imaging can cover a wide FOV of up to 200° [4,5] and still achieve a relatively high spatial resolution, this is not usually the case for OCT volumes. Raster scans of most commonly used commercial OCT devices typically cover a FOV between 20°× 20°and 30°× 30°. The reason for this relatively small FOV is a trade-off between 3 factors: Acquisition time, spatial resolution and FOV.

Acquisition time of a scan should be as short as possible to minimize the negative influence of involuntary eye movement and usually lies in between 2 to 4 seconds. If one wants to increase the FOV while keeping the spatial resolution fixed, this can only be achieved by increasing the acquisition speed of the scanner which comes at the cost of lower sensitivity. A lot of research focuses on creating OCT devices with higher scanning rate in order to create those larger FOVs, with some Swept-Source (SS) OCT devices managing A-Scan rates up to the MHz range and a FOV of up to 100°× 100° [6–8]. SS-OCT for ophthalmic imaging mainly uses imaging light in the 1060 nm wavelength band which provides better penetration through the RPE and into the choroid. However, the image resolution (both axial and lateral) is typically lower than in spectral domain systems operating in the 840 nm band [9]. An important extension of OCT, OCT angiography, requires multiple scanning of the same location in combination with a dense sampling of the retina with high sensitivity in order to visualize microvasculature [10]. This is typically associated with a small field of view of about 20°× 20°and for a more complete assessment of the disease the demand for image stitching is high [11]. In research there are often very specific requirements for OCT systems that can limit ones choice with regards to the technologies one can use. Examples are, adaptive optics (AO) OCT which - apart from a high imaging speed - usually requires a complex lens and mirror system, providing a very high spatial resolution but at the cost of a severely limited FOV of only up to 2°× 2° [12], or visible light OCT which allows quantification of blood oxygenation in retinal vessels [13], but for which no useful swept source exists. Polarization Sensitive (PS) OCT [14,15], a functional extension of standard OCT imaging, often require high spatial resolution in order to visualize detailed structures such as individual nerve fiber bundles which comes at the cost of a comparatively small FOV 10°- 20°. Furthermore, the quantitative measurement of PS-OCT data like retardation and optic axis orientation require sufficient signal to noise ratio which can require repeated scans to generate high-quality averaged data sets [15,16], therefore demanding a method to register and stitch such data sets.

Considering an OCT acquisition time of a few seconds, motion artefacts are an inevitable consequence due to involuntary eye movement [17]. A lot of work has gone into motion compensation and image registration which can broadly be divided into prospective and retrospective approaches. Retrospective approaches try to find spatial correspondence between voxels of multiple volumes - often using additional data such as an SLO image as a reference image [18,19]. Considering that retinal motion artefacts in OCT data are present mainly along the slow scanning axis, special scanning patterns can also help to provide data that can more easily be motion compensated even without a motion free reference image. Such methods include acquisition of multiple (at least 2) volumes - swapping the fast scanning axis in between acquisitions [20,21] or using the Lissajous scanning pattern that features elliptical scanning beam patterns of different frequencies to record elliptical B-Scans that can be registered against one another using mutual information to correct for motion [22]. One major flaw in these retrospective motion compensation methods, however, is that they can lead to missing data when shifting parts of it to correct for detected motion. Thus, prospective methods for compensating retinal motion are preferred. These systems rely on an additional imaging device (often an integrated SLO) that can be used to track retinal movement in real time. Some tracking algorithms use only parts of the image that have to be selected manually by the operator [23], which limits their potential use and makes their performance very dependent on the region selected by the operator.

In this paper we address the problems associated with a small FOV by proposing a novel method that allows to take intra acquisition movement compensated projection images acquired from OCT devices of any scanning technology to create large FOV projection images from volumetric OCT data. We demonstrate the performance of the method for image data acquired with a PS-OCT instrument in healthy and diseased eyes.

## 2. Methods

### 2.1 PS-OCT system

The imaging system used was a custom-built prototype PS-OCT operating at 860 nm with an A-scan rate of 70 kHz and a FOV of 28°× 21°(1024 A-Scans × 250 B-Scans), an integrated Line Scanning Laser Ophthalmoscope (LSLO) for tracking purposes operating at 790 nm with a refresh rate of 60 Hzand a line width at the retina of 20 μm [23]. The axial and lateral resolutions of the system are 4 μm (in tissue) and 17 μm ($\frac{1}{e^{2}}$ intensity full width), respectively [24]. The lateral pixel spacing (assuming that 1°scanning angle corresponds to 300 μm on the retina) is 8.2 μm (x) and 25.2 μm (y). The system is controlled via a custom-built LabView software that allows arbitrary positioning and movement of the OCT scanning beam in X and Y axis [25]. Additionally large FOV fundus photos (45°× 45°) were acquired using a separate Nonmyd WX-3D fundus camera (KowaCompany, Japan).

For some clinical applications, the supported FOV of the OCT instrument is too small. In order to overcome this obstacle we decided to acquire volumetric OCT scans at several different fundus areas and then stitch them together to create one large FOV scan. The main steps required for this method to work are first acquiring OCT scans at several fundus areas. These scans should be free from movement artefacts, as these would not only affect the final result but potentially reduce the quality of the stitching. Once OCT scans with sufficient quality have been acquired they will need to be stitched together whilst compensating for optical distortions. These problems - and our proposed methods for solving them - will be discussed in the subsequent sections.

### 2.2 Improvement of the retinal tracker

**Fig. 1.**
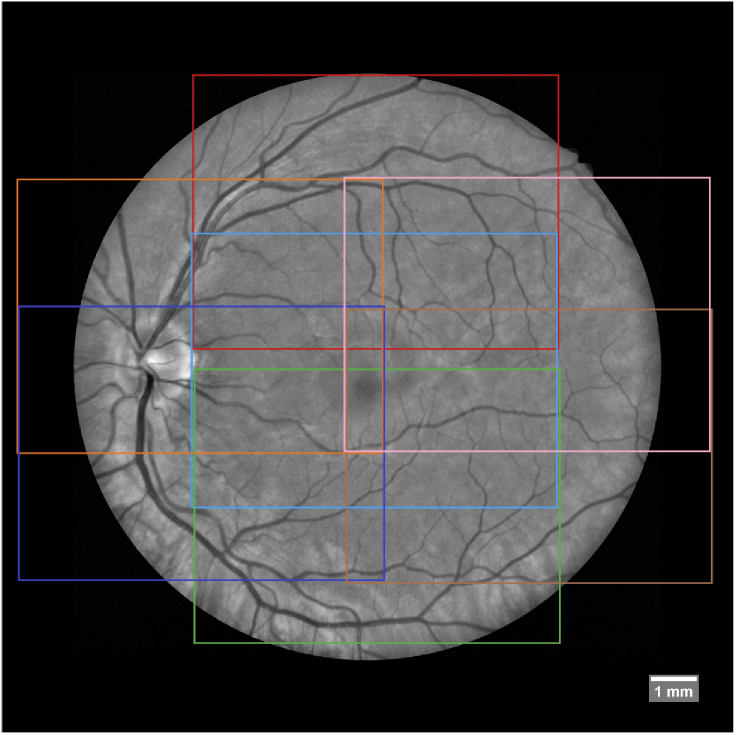
The 7 retinal positions (indicated by the colored rectangles) that have been imaged with PS-OCT to cover a large FOV overlaid to the green channel of a fundus photo.

In order to cover a wide FOV, PS-OCT volumes at 7 retinal positions are acquired. These retinal positions with respect to the fovea are central (centred around the fovea), central superior, central inferior, temporal superior, temporal inferior, nasal superior and nasal inferior, with neighbouring regions overlapping (Fig. 1). The chosen scanning pattern covers a FOV that is as large as possible with the least amount of scans considering the maximum eccentricities of the fixation locations that are available by the internal fixation target of the system. This results in a large overlap between neighbouring regions that reaches roughly a third of each volume. To achieve a higher signal to noise ratio on the final result, 3 OCT volumes are acquired for each of these regions, resulting in a total of 21 PS-OCT volumes per subject.

Although the used PS-OCT device incorporated already retinal tracking modifications were necessary to allow faster and more robust OCT data acquisition. In the original tracking software, the operator had to manually select a feature rich position in the SLO image, typically a retinal vessel crossing or bifurcation which is quite time consuming [23]. This approach works fine for scans in the fovea region where the expected movement is not too large and where feature rich structures (as vessel crossings) are present in the SLO image.

Problems, however, occur in peripheral (temporal) scan regions where only few vessel bifurcations are present or when the selected reference region is no longer visible - either because of movement putting it outside of the FOV, or because of reflection artefacts originating from the cornea (to be discussed in 2.2.1) that are overlapping with the reference region. To overcome this limitation we developed a new intra volume scanning live tracking software using a 2D phase correlation approach that utilizes the entire SLO image information and does not require any operator interaction for the tracking. Phase correlation is widely used in image tracking and compared to cross-correlation features a much more distinct peak at the point of registration [26]. Additionally, it is more robust against changes in illumination or average intensity - making it our method of choice.

#### 2.2.1 Preparation of SLO images for tracking

**Fig. 2.**
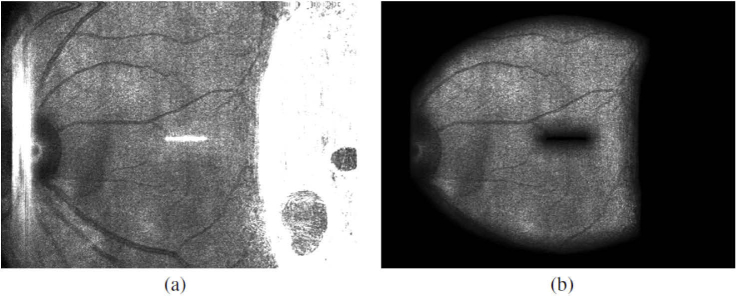
SLO image showing various reflection artefacts (a) and after all artefacts are masked out (b).

2D phase correlation uses the frequency components attributed to edges in an image to find the correct spatial offset between two images [26]. This means however, that any and all edge-like features in an image contribute to that correlation. It is thus important to make sure that only edges corresponding to anatomical features like retinal vessels remain in the image and edges caused by any other artefacts are filtered out. As can be clearly seen in Fig. 2(a) the SLO fundus images of the device contain some distinct artefacts, mostly caused by reflections of the imaging light at lens interfaces of the system and at the cornea of the imaged subjects. Since these artefacts could be interpreted as features by the tracking algorithm they need to be excluded from the image. Note that the last telescope of the LSLO is shared with the OCT beam. Thus, hardware methods for artefact removal such as implementation of a slit or the use of polarization optics cannot be directly implemented into the system. The bright elongated structures in the centre and on the left side in Fig. 2(a) are caused by reflections within the optics of the SLO beam propagation and they remain rather constant. The large bright saturated area on the right hand side of the image, however, is caused by a reflection at the subjects’ cornea and will vary in position depending on the illumination angle. This artefact often covers up to a third of the SLO image. Since the artefacts feature a much higher intensity than the rest of the SLO image these can be easily detected via thresholding. Given a SLO frame I(x,y) a mask M(x,y) containing the artefacts can be created via

(1) $$M(x,y)=\left\{ \begin{array}{ll} 0 & \text{if }I(x,y)<t\\ 1 & \text{if }I(x,y)>=t \end{array}\right.,$$

with threshold value t being empirically chosen based on a histogram analysis of I to best encapsulate the artefact areas. To eliminate small patches in the mask caused by noise the mask is further processed using morphological operators. The resulting mask CM(x,y) is created via

(2) CM(x,y)=((M(x,y)⊕S)⊖S)⊕T,

with S and T both being binary circular masks used for morphological operations as denoted by dilation ⊕ and erosion ⊖ operator. S is a relatively large mask used for morphological closing, whilst T is comparatively smaller as it is used to artificially enlarge the masked region as is needed for the following step.

Since we are using a phase correlation approach for tracking which requires Fourier transforming the image we have to deal with the image edges as the Fourier transform expects the image to be composed of periodic signals. Thus we apply a Hamming window H(x,y) to the image to avoid creating artificial edges at the border of the mask [27,28] and the mask CM(x,y) is smoothed with a sliding window average filter to get a continuous transition between masked and unmasked regions. Both the CM(x,y) and H(x,y) are applied to I(x,y) to obtain a filtered image F(x,y) via

(3) F(x,y)=I(x,y)∗MIN((1−CM(x,y)),H(x,y)).

The result of this preprocessing can be seen in Fig. 2(b).

#### 2.2.2 Intra volume-scanning live tracking

The intra volume-scanning live tracking itself consists of several phases. When starting a PS-OCT volume acquisition at a specific image location a training phase is initiated. During this phase 20 consecutive frames of the SLO camera are recorded and preprocessed as presented in section 2.2.1. The specific number of frames empirically yielded the best results in terms of tracking performance and short training phase time. One out of the 20 frames is selected as a reference image for registration. To find the best candidate the images are sorted by the number of usable pixels PC of the mask CM(x,y) from Eq. (2) via

(4) $$PC=\sum_{x=0}^{X}\sum_{y=0}^{Y}\left(1-CM(x,y)\right),$$

with X and Y being the dimensions of the image. The frame with the highest amount of usable pixels is selected as a base reference image and the next 9 frames (in descending order of usable pixels) are registered to that base frame using 2D phase correlation. After registration and correcting for offset in X and Y dimension the 10 frames are averaged as

(5) $$RF(x,y)=\frac{\sum_{n=0}^{9}I_{n}(x,y)}{10},$$

with $I_{n}(x,y)$ being the $n^{th}$ used image. The new averaged image RF(x,y) is then pre-processed based on the methods in section 2.2.1 to serve as the reference image for live-tracking since it now features a higher signal to noise ratio (SNR) compared to single SLO frames, without adding significant motion blur due to the registration process - resulting in a much more robust live-tracking. This training phase can also be repeated at any time between measurements.

After the training phase is completed the automatic tracking begins. Given 2 images - f(x,y) and g(x,y) being the reference image and the current preprocessed SLO frame respectively - and F(ω) and G(ω) their respective Fourier transforms, we can get the normalized cross spectrum R(ω) of f and g via

(6) $$R(\omega )=\frac{F(\omega )G^{*}(\omega )}{|F(\omega )G^{*}(\omega )|+\epsilon },$$

with ϵ being a small value added for stability. To increase robustness of the tracker we first have to high-pass filter our images since we are only interested in tracking sharp edges which translate into high frequency parts in the Fourier transformed image. High-pass filtering in frequency space is a simple multiplication, so given a Fourier transformed image F(ω) and a Gaussian kernel K(s) of size s centred in an image the same size as F(ω) the filtered image FF(ω) can be obtained via

(7) FF(ω)=F(ω)∗K(s).

By applying this step twice to the reference image we can perform it before the acquisition loop starts and consequently save computation cost during the acquisition. So by instead using

(8) FF(ω)=F(ω)∗K(s)∗K(s),

we can modify Eq. (6) to

(9) $$R(\omega )=\frac{FF(\omega )G^{*}(\omega )}{|FF(\omega )G^{*}(\omega )|+\epsilon }.$$

**Fig. 3.**
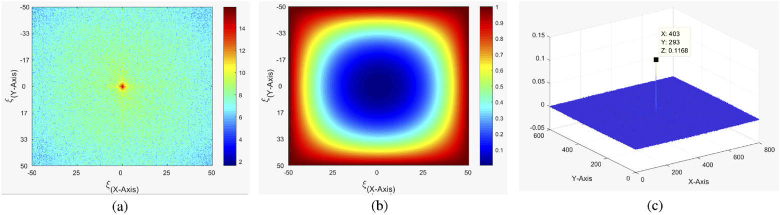
Magnitude of the Fourier transformed SLO frame. Colorbar displays log of the magnitude and spatial frequency is given in cycles per mm (a). High-pass filter indicating multiplication factor for each frequency - this is directly multiplied with the Fourier transformed reference image (b). Phase correlation peak between reference image and another SLO frame. The offset from the center point (coordinates indicated in X: and Y:) denotes the observed translation (in Pixel) between the scans. The peak can be clearly distinguished from the background noise (c).

After applying the inverse Fourier transform r(x,y) of R(ω) a clear narrow peak can be detected at the (x,y)-position in the spatial domain indicating the spatial offset between the reference image and the current SLO frame as can be seen in Fig. 3. The peak location in the spatial domain is detected by searching for the location with the maximum value in r(x,y) and the detected offset is passed to the OCT scanners to compensate for motion. When tracking using this setup, however, we noticed a jittering effect in the scanner motion when trying to follow the detected movement. This happened due to actual offsets not being limited to integer values for pixel coordinates but any numbers in between. So in case of an offset lying between two neighbouring pixels the algorithm could sometimes rapidly alternate between those two numbers from frame to frame. To solve this the image r(x,y) was first smoothed with a 3x3 Gaussian filter and after first finding the offset based on the maximum peak in the image, the true offset in between pixels was estimated using neighbouring pixels as weight. This estimated offset in X and Y axis, as well as the peak height of the registration are delivered to the OCT scanning head controller abstracted in software by a LabView component. Here the pixel offset values are converted to X- and Y-scanner voltage offsets using a pre-determined conversion factor. This factor was determined in a model eye by manually tilting the model eye and adjustment of this factor until the original location of the retina (before the tilt) is imaged again through the application of the tracking offset. Before correcting for movement however, two things are considered: the offset value and the peak height. As for the offset there is a fixed limit on the maximum allowed distance that the scanner may move within the timeslot between two frames. This is implemented as a safety feature as to not damage the equipment with rapid large movement. The peak height threshold however is dynamically calculated taking the average peak height of the past 60 valid frames in order to ensure that the registration was indeed successful. Should any of these two conditions not be met, the frame is not tracked and instead a retake phase is triggered.

The most likely scenario to enter a retake phase is due to a blink or very large eye movement. For a SLO frame taken at time $t_{n}$, a retake phase will be triggered if a large eye movement happened between $t_{n-1}$ and $t_{n}$. Blinks, however, are slightly more complicated. It takes on average about 110 ms for the eye lids to be completely closed after a blink has been initiated [29]. This corresponds to about 6 frames of the live SLO image. Since the phase correlation algorithm can cope with only a small area of the SLO image actually carrying information, the eye may have already almost completely been closed by the time the algorithm registers it as a blink, meaning that only the last 2-3 B-Scans could have already been compromised by the blink. Thus when entering the retake phase the scanner not only retakes the position where the phase has been initiated at, but instead moves back a number of B-Scan positions to resume the acquisition from there on - making sure to rescan everything that could have been compromised by the blink. After empirically testing this setting with numbers between 1 and 10 it was found that moving back 4 B-Scan locations was the smallest amount that still prevented corruption from blinks most of the time. Once the scanners moved into position the system waits for SLO frames to return valid values (i.e. values that would not trigger retake phase), and once received, continues acquisition, overwriting the corrupted B-Scans in the volume under acquisition.

### 2.3 Combining individual en-face projections into one large FOV projection

After the acquisition of motion compensated OCT data for all 7 imaged fundus areas (cf Fig. 1) we can now focus on combining that data in order to cover one large FOV image of the fundus. To accomplish this, first we need to find coordinate system transformations for the spatial domains of each OCT data set recorded at different fundus areas which translates every data location of one OCT data set to its corresponding position in the large FOV data representation of the fundus. Applying these transformations in volume/image fusion is the second step to build up one common large FOV data set.

This problem is similar to the tracking problem discussed in section 2.2.2. We want to know the displacement values of data from one retinal location with regards to data from another, both representing the same anatomical position in the subject. As volumetric OCT data is available, we have the choice of using registration techniques in 3 dimensions and thus registering whole volumes, versus registration in 2 dimensions, using only data of a 2D projection of the volume. As in many applications the generation of en-face images is the final outcome, we opted for using a 2D registration approach.

The next decision is about which registration technique to use. The two major methods we considered were Feature Point Detection and Phase correlation as we already used for tracking. In Feature Point Detection a specific kernel is applied on the image in order to detect features of interest. Different kernels will detect different types of features and should be chosen according to the individual requirements. Typically, large corners or very distinct shapes indicate a strong feature for most common kernels. Feature points are extracted from both images and after applying a RANSAC algorithm for filtering out weaker features they are compared between the two images [30,31]. Matching feature point pairs are then used to estimate a transformation to correct the offset between the two images. In our case however, there are some problems with this approach. The most distinct feature in retinal intensity projection images is the retinal vessel tree. When trying the common method for feature detection (Speeded Up Robust Features - SURF) we quickly realized that most feature points were detected at vessel crossings and bifurcations and only very little else. This meant that some regions in the image had vast amounts of data points while the rest featured almost none which could skew the perceived distortion of the image. Additionally we often found bad matches of feature points between different intensity projections and/or the fundus image. Hence we chose a 2D phase correlation approach using the wide field fundus image as reference. What follows are the individual steps taken in more detail.

#### 2.3.1 Preparation of the fundus photo as reference image

As reference image we acquired a wide field retinal fundus image with a FOV of 45°centred around the fovea. The image is rescaled such that a distance of 1 Pixel (=8.2 μm) in X or Y axis corresponds to 1 Pixel in the PS-OCT Data in X or Y axis. Our fundus photos were colour images featuring RGB values. Since the green channel showed the highest contrast with regards to retinal vessels we used that channel and discarded the other two. Finally zero padding was added around the borders because our stitched projections will cover a slightly wider FOV than the fundus photo provides (as was shown in Fig. 1).

#### 2.3.2 Generation of en-face projection image

**Fig. 4.**
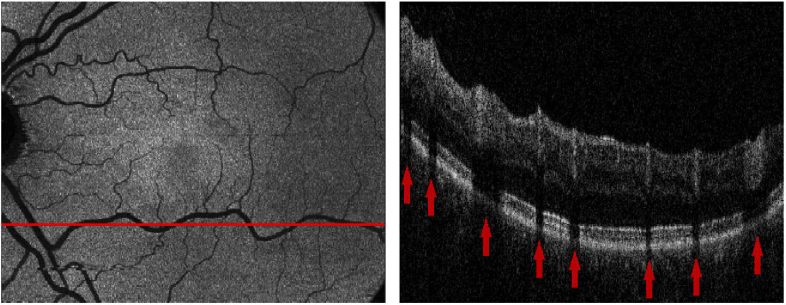
Left: Intensity projection image over the RPE band. Right: Cropped OCT B-Scan at position indicated left. Red arrows mark the location of shadows caused by retinal vessels.

As a first step 3D PS-OCT image data are calculated from the acquired data sets, providing intensity, retardation and birefringent axis orientation, including a compensation of the corneal birefringence, based on the methods described in [32–34]. Next, intensity projections are obtained by summing up the values in depth (axially) calculating a fundus-like en face image based on OCT volume data (set of B-Scans). The contrast of the vessels can however be greatly improved by limiting the interval of summation to the retinal pigment epithelium and photoreceptor bands (RPE) as shadows caused by the vessels have the highest contrast in this area (Fig. 4). For this the RPE layer will be segmented from the volume, followed by a flattening of the volume according to the RPE layer and then a specified region in depth over which we sum the intensity values will be taken.

**Fig. 5.**
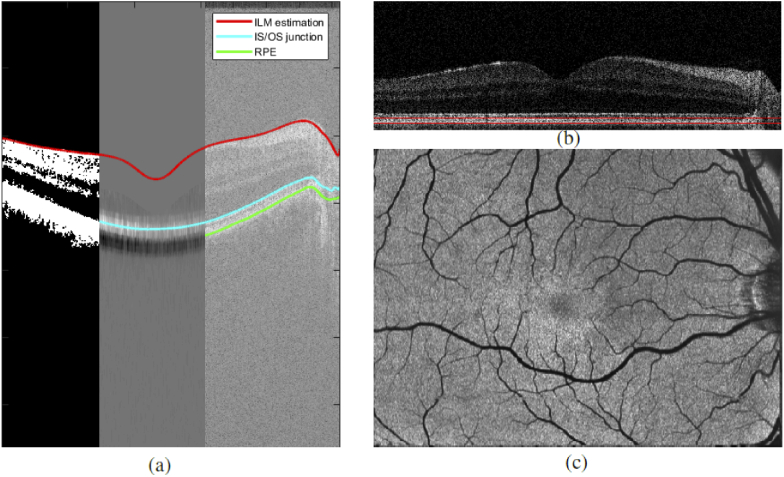
Path to high contrast intensity projection images. (a) shows the intermediate segmentation steps. left: ILM estimation via thresholding; middle: IS/OS detection via local gradient; right RPE detection via DOPU image. (b) shows the B-Scan flattened at the RPEwith the 30 μm thick band for intensity projection image generation drawn in red. (c) shows the final averaged intensity projection image of a scanning region.

Detecting retinal layers can be seen as an optimization problem which we tackle using a graph-based shortest path solution on a per B-Scan basis, similar to the implementation described in [35]. The steps needed for segmenting the retinal layers are as follows. Given an intensity B-Scan image I(x,z) the thresholded image It(x,z) is calculated via

(10) $$It(x,z)=\left\{ \begin{array}{ll} 0 & ifI(x,z)<t(x)\\ 1 & ifI(x,z)>=t(x) \end{array}\right.,$$

with t(x) being the average of the 512 highest values for each X position (=A-Scan) of the image I(x,z). Next, the thresholded image It(x,z) is smoothed via morphological closing using a small disk and binary patches below a certain size are removed to avoid "blobs" of noise above the inner limiting membrane (ILM). The path of the ILM is then estimated by taking the top most segmented pixel for each X position and smoothing the result with a 101 Pixel wide sliding window average filter to eliminate outliers (as seen in Fig. 5(a) left). When looking at a B-Scan from top to bottom it can be seen that both the ILM and the inner segment outer segment (IS/OS) junction have a very clear transition from dark to bright which results in a high value gradient when using a large kernel. Considering that the retinal layers will never cross each other we can make use of the positional information of the ILM and set all values of the gradient image above the ILM (and up to half the size of the gradient kernel below the ILM) to zero. This way only the gradient information of the IS/OS junction remains which can then be used as weights for the shortest path segmentation (see Fig. 5(a) centre). For detecting the RPE we make use of the polarization sensitivity of our OCT system. Since the RPE layer is a depolarizing structure the degree of polarization uniformity (DOPU) will have a significantly lower value than other retinal layers [14,36]. Thus we calculate the inverse DOPU image (since we want the weights for the shortest path to be high on the RPE) and eliminate everything above the IS/OS boundary before applying the shortest path search (see Fig. 5(a) right). In principle the RPE can be segmented using the DOPU image without first calculating the other 2 retinal layer boundaries [36]. On very low quality OCT scans however it can occur that the polarization data is very noisy which in turn affects the DOPU image. This can potentially cause the graph search algorithm to jump out of the RPE and into other retinal layers if it is not preliminarily bounded to that region. By first segmenting the ILM and IS/OS boundary as a region limitation we can even further reduce the chance of segmentation errors. Finally the B-Scan is flattened using the height information of the RPE and the two boundaries seen in Fig. 5(b) are used as upper and lower limit for summing up the intensity values of the RPEover a height of 30 μm to create an intensity projection image. Every image generated from each volume scan is visually inspected for severe motion artefacts and only images that passed this inspection are rigidly registered to each other using the methods described in section 2.2.2. Then the images are averaged to create one noise reduced en-face projection image per imaged region as can be seen in Fig. 5(c).

#### 2.3.3 Registration via pyramid approach

**Fig. 6.**
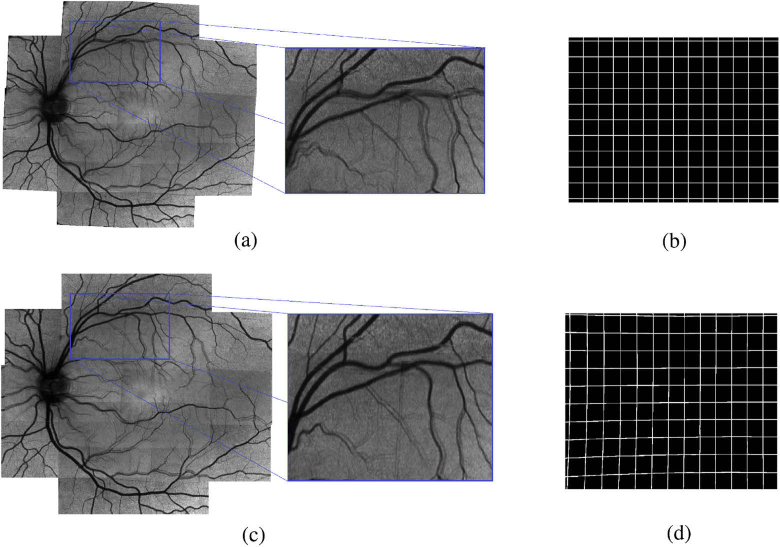
Left Column: Stitching considering rotation and translation of the intensity projection images without (a) and with optical distortion compensation (c); Right Column: Regular Grid (b), effect of optical distortion compensation shown on b (d).

**Fig. 7.**
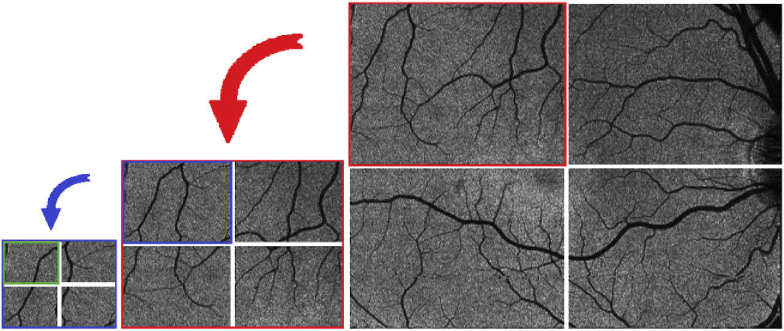
Cut of the OCT projection image into 4 quadrants shown for one branch of 3 level recursion.

Consider the two images B(x,y) and M(x,y) being the wide field fundus image as reference and one of the calculated intensity projection images, respectively. We want to find a geometric transformation T(M) that registers M(x,y) to B(x,y). As mentioned in the beginning of this section we chose a 2D phase correlation technique as already presented in section 2.2.2. Unfortunately a simple global rigid transformation model does not regard for all optical distortions present in the image. Both, the fundus photo and OCT projection images are acquired at slightly different retinal positions using different devices, optics and entry angles. This causes all of the images to have slightly varying optical distortions mainly caused by non-perfect optics of the system and the cornea, lens and shape of the eye. Thus even when finding the correct global translational offset between images these will not perfectly line up due to their different optical distortions as can be seen in Fig. 6(a). To reduce the effect of optical distortions we performed a pincushion transformation on the projection images. The model for distortion [37] is

(11) $$r_{c}=r_{u}(1+kr_{u}^{2}),$$

with $r_{c}$ and $r_{u}$ being the normalized corrected and uncorrected distance to the centre, respectively, and k being the distortion factor which depends on the optics of the system. Figure 6(b),d shows the transformation effect on a synthetic grid. The value for k was obtained by applying the transformation on a set of healthy subjects with varying values and picking the one that resulted in the lowest average variation between transformed OCT projection image and fundus photo. As seen in Fig. 6(c) this visibly improves the result, but it still leaves notable distortion errors since this simple transformation cannot compensate for all the subject specific sources of distortion. The remaining distortion can then be compensated using a second order polynomial tranformation function in the form of

(12) $$f(x,y)=ax^{2}+by^{2}+cxy+dx+ey+f,$$

with x and y representing the coordinates of the image to be transformed and a to f being coefficients which are estimated using a MatLab built-in estimator based on the methods of [38,39]. For this however, we first need a number of point pairs indicating the transposition of several points in one image to the reference image. Automatic feature point detection methods - as discussed in the beginning of this section - would provide a number of point pairs needed for this, but for the reasons already mentioned we backed away from using this method. Additionally we would not have a great influence on the location of these feature points which could cause some regions in the image to be greatly over-represented while others may be under-represented, potentially reducing the quality of the transformation. Instead we chose to keep our phase correlation method as a template based matching method to determine the required set of point pairs. The phase correlation method had already proven itself to be very robust and accurate (see section 3.1) and we simply split up our projection image into multiple parts. In theory one can do a simple split of the image into m∗n non-overlapping parts and perform the phase correlation on each of those parts separately in order to end up with m∗n equally spaced feature point pairs that can be used for estimating the parameters of the transformation function. A higher value for m and n would then result in more point pairs which is good for the parameter estimation, it would however also reduce the size of the individual image parts and in turn greatly reduce the robustness of the phase correlation. To avoid this problem while still increasing the amount of image parts we chose a pyramid-based approach for registration which works as depicted in the following pseudocode. The function takes as input $I_{fundus}$ and $I_{proj}$ which are the fundus photo and the current OCT projection image that is to be registered, respectively. Additionally there is a pixel mask $I_{mask}$ which is used to limit the search region of the registration peak to an expected region. ${\vec{v}}_{cent}$ indicates the center coordinates of the current projection part in respect to the entire OCT projection image. n indicates the image partitioning levels that should be performed. After performing phase correlation between fundus photo and OCT projection image the result is multiplied componentwise with the mask $I_{mask}$ and the coordinates of the highest peak in combination with the ${\vec{v}}_{cent}$ is stored as a point pair. Now the image $I_{proj}$ is split into 4 quadrants ($I_{subim}$) as illustrated in Fig. 7 and the $I_{mask}$ for each $I_{subim}$ is constructed. This mask $I_{mask}$ is basically a 2D Gaussian shape, half the size of $I_{subim}$ and centered around the newly found peak from the registration (${\vec{v}}_{cent}$), compensated for the relative offset between the quadrant center location in respect to the current projection image center. The set of point pairs for the $I_{subim}$ are calculated by applying our function estimatePointset recursively until the image partitioning level n reaches 0.

When calling the function an $I_{mask}$ has to be provided. If there is external prior knowledge regarding the expected location available (eg. knowing the rough retinal position of the scan) it can be created manually - otherwise we simply set all pixels in $I_{mask}$ in a neutral manner to 1. After obtaining the list of point pairs R the 30% with the weakest correlation peak are dismissed to avoid inaccuracies caused by potentially wrong registration. The remaining point pairs are used to estimate the parameters of the second order polynomial (Eq. (12)) which can then be used to transform OCT projections to their respective positions in the wide FOV image.

**Algorithm 1:** pyramid-based registration

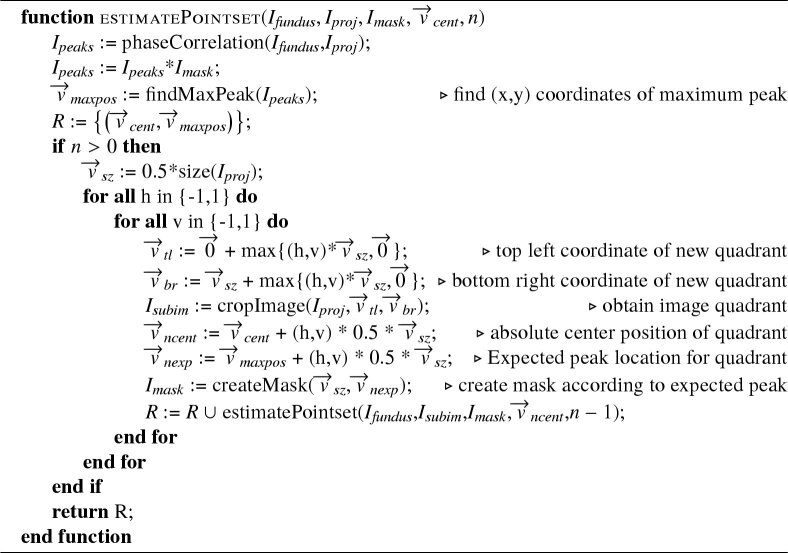

#### 2.3.4 Stitching projections

Given a projection image $I_{n}(x,y)$ as the $n^{th}$ acquired OCT projection and its transformation function $f_{n}(x,y)$ as obtained through the pyramid based registration we can now obtain our large FOV projection image. Since there will be multiple data points (from the repeated and partially overlapping volume scans per position) for most of the (x,y) positions it makes sense to not directly transform the individual projections to one final large field image, but instead create one separate large field image for every acquired projection and only after transforming average over that via

(13) $$I_{Ln}(x^{\prime },y^{\prime })=I_{n}(f_{n}(x,y)),$$

with $x^{\prime }$ and $y^{\prime }$ being given for each n by

(14) $$(x^{\prime },y^{\prime })=f_{n}(x,y).$$

We cannot however average $I_{Ln}$ over all n directly, since the number of observations will vary along coordinates. Thus we first need to create a custom divisor based on that number

(15) $$div_{n}(x^{\prime },y^{\prime })=\left\{ \begin{array}{ll} 1 & \text{if }f_{n}^{\prime }(x^{\prime },y^{\prime })\text{ within projection boundaries}\\ 0 & \text{otherwise} \end{array}\right.,$$

(16) $$div(x^{\prime },y^{\prime })=\sum_{n=1}^{N}div_{n}(x^{\prime },y^{\prime }).$$

Now a correctly averaged large field projection image can be created using

(17) $$I_{L}(x^{\prime },y^{\prime })=\frac{\sum_{n=1}^{N}I_{Ln}(x^{\prime },y^{\prime })}{div(x^{\prime },y^{\prime })}.$$

This will, however, cause visible stitching seams caused by slight variations in illumination between the individual projection images, as well as by the fact that our OCT volumes sometimes suffer from poorer quality near the borders of the image. To counter that we will introduce one more final transformation to the projection images $I_{n}$. In order to obtain a smooth transition we will not average directly over all N observations but instead introduce a weighted average, taking into account how close to the edge a specific pixel on a projection image is. The closer to the image border, the less weight it should possess. An easy way to achieve that is to apply a slightly modified Hamming window to each image $I_{n}$ prior to transformation. For this we will also have to change our divisor div. Thus we will change Eq. (13) to

(18) $$I_{Ln}(x^{\prime },y^{\prime })=(I_{Hn}(f_{n}(x,y)),$$

with $I_{Hn}$ being the $n^{th}$ projection after applying a custom Hamming window, and we will modify Eq. (15) by using

(19) H(x,y)=h(x,y),

with h(x,y) being the Hamming window multiplication value and to have

(20) $$div_{n}(x^{\prime },y^{\prime })=\left\{ \begin{array}{ll} H(x,y) & \text{if }f_{n}^{\prime }(x^{\prime },y^{\prime })\text{ within projection boundaries}\\ 0 & \text{otherwise} \end{array}\right..$$

**Fig. 8.**
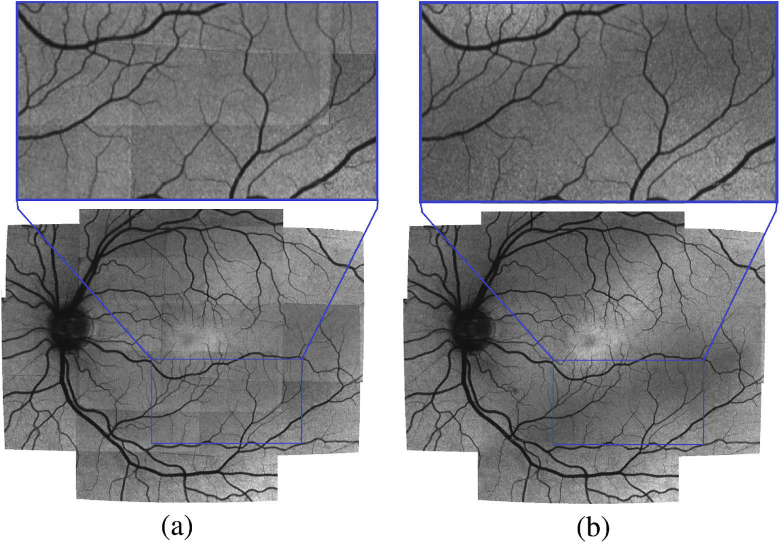
Stitched large field intensity projection (bottom) with zoomed in region (top). The standard averaging approach leaves visible seams at the edges of the individual images used (a) while the adaptive weighted method does not show such seams (b).

The equations Eq. (16) and Eq. (17) remain unchanged. Figure 8 shows the difference of using weighted averaging compared to the standard approach.

### 2.4 Measurements

The study was approved by the university’s ethics committee and is in agreement with the tenets of the Declaration of Helsinki. All subjects measured were healthy volunteers and glaucoma subjects and we obtained informed consent. Six healthy subjects aged 27 to 63 years (mean 35) were imaged for testing retinal tracker accuracy. Five healthy and five glaucoma subjects aged 25 to 48 years (mean 39) and 46 to 71 years (mean 56) respectively, were used for measuring accuracy of registration and stitching. The glaucoma data was taken from an ongoing study planned to finally include a total of 100 patients with early glaucoma.

In order to meet the real time constraints that a retinal live tracker imposes the algorithm was implemented on a GPU (Nvidia GeForce GTX 1070TI) using the CUDA application programming interface in C++. It was embedded in the instrumentation software of our PS-OCT device as an additional module in the LabView environment. The processing time for each SLO frame was between 4 and 5 ms. With the SLO imaging rate of 60 Hz we get one frame every 1.14 B-Scans. This means that, assuming an average processing time of 4.5ms, the response time of the tracker can range from 4.5ms up to 21.2ms (processing time + maximum time delay between SLO frames at 60 Hz). In reality the system response time will most likely be between those two extremes, averaging to about 12.85ms.

The registration and stitching algorithm was implemented using both MatLab (MatLab R2017b Mathworks Inc.) and CUDA and performs in about 1 minute per subject. This time requirement could still be significantly reduced by porting more of the calculation intensive functions to the GPU.

Over all the scanning process of acquiring 21 volume scans including subject alignment takes about 30 minutes using our system setup. The time required to evaluate the raw data and generate a stitched large FOV projection as presented in section 3 is about 1 hour.

## 3. Results

### 3.1 Tracking

The accuracy and robustness of the modified tracking method were evaluated as follows.

**Fig. 9.**
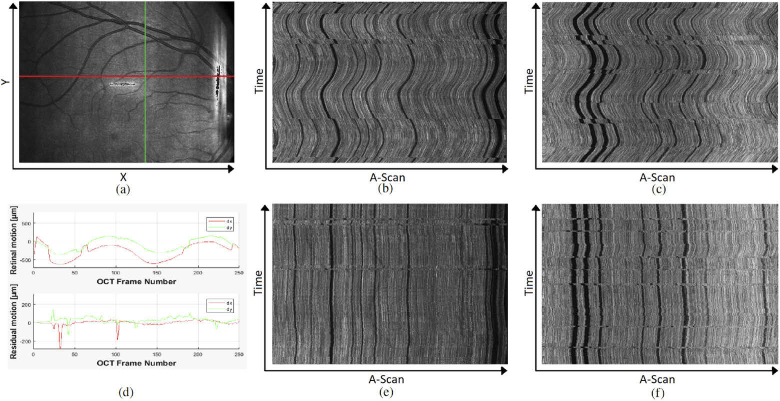
SLO image indicating the scanline of the repeated horizontal (red) and vertical (green) B-Scan (a); En-face projection (over RPE) of horizontal and vertical repeated B-Scan without tracking (b and c respectively) and with tracking (e and f respectively); Graph displaying the retinal motion of the uncompensated projections (d top) and the residual motion when using the retinal tracker (d bottom).

**Fig. 10.**
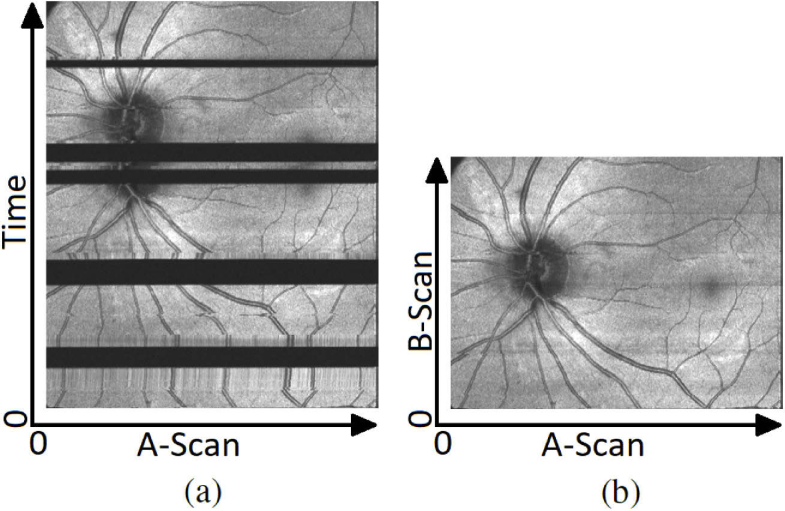
Projection of the volume scan with tracking and blinking compensation turned on. (a) Data set without removal of blinks and poor tracking, (b) Stored data set with automatically discarded artefacts and re-scanned locations. Some small residual artefacts can still be detected - especially the thick vessel near bottom left - this will be further analyzed in section 4

Using a scanning protocol that acquires horizontal B-Scans at the same retinal Y-position 250 times, we performed the measurement centred at a retinal location that featured a retinal vessel crossing perpendicular to the scan region. Whilst performing scanning of the 250 B-Scans, the subject had to fixate on a target that continuously oscillates with constant speed along the horizontal B-Scans scanline such that the actually measured B-Scan location would also move along the horizontal axis which can be seen in Fig. 9(b). This measurement was repeated with live tracking activated to counteract the retinal movement (Fig. 9(e)). To also assess the accuracy on the Y-Axis we applied our evaluation method with vertically oriented B-Scans at constant X-position, without and with tracking (Fig. 9(c),(f) respectively). Tracing the offset of a vessel in the resulting intensity projection images across individual B-Scans, we can detect how much motion is present per axis. Figure 9(d) shows the uncompensated motion (top) and the residual motion while using tracking (bottom). These paths were obtained by first performing a rough estimation by manually marking positions along a vessel with high contrast and then using this path as a rough bias for the subsequent cross correlation between each B-Scan, utilizing one manually selected B-Scan as reference. When looking at the intensity projection with tracking on some semi-periodic artefacts can still be observed. These artefacts are caused by microsaccadic eye movement that are too fast for the retinal tracker to be fully compensated. These positions can also clearly be observed in the residual motion graph in Fig. 9(d) as sharp peaks. Table 1 summarizes the results of the motion compensation averaged overthese 6 healthy subjects. It includes the maximum range of residual motion as well as the standard deviation thereof. The analysis was performed for all 250 B-Scans, as well as only for the 150 best matching ones in order to obtain performance measures excluding microsaccadic artefacts. When excluding microsaccadic artefacts the range of residual motion for both X-, and Y-Axis are at about 5 and 6 Pixel respectively (10 μm = 1 Pixel). Considering that this is tested with a moving fixation target, residual motion is expected as any tracking will be slightly behind. The standard deviation of about 1.5 Pixel for both axes is close to the limit of what can be spatially resolved. For testing robustness of the retaking function we intentionally introduced several artefacts during acquisition of a PS-OCT volume to see whether accuracy of the registration would be affected. These artefacts include wandering reflexes as well as intentional blinking and large eye movements during acquisition. To demonstrate the robustness we temporarily saved two volumes per scan - one where blinking compensation overwrites the saved volume at the correct position and one where B-Scans recorded for artefact correction are simply appended to the volume. The latter is basically indicating the position of the scanner over time. Figure 10(a) shows this raw acquisition - blinks can be clearly seen as dark horizontal blocks since there is no signal at the corresponding position. After each blink it can also be seen that a small area prior to where the blink occurred is being measured again before continuing normally (due to the retake function going back an additional 4 B-Scan positions). In Fig. 10(b), however, these artefacts have been removed, even though multiple blinks had occurred during acquisition.

**Table 1.** Residual motions in X and Y Axis detected in OCT B-Scan images recorded with retinal tracking and moving fixation target

Moving Axis	X-Axis		Y-Axis	
	SD	Range	SD	Range
Residual motion ( μm)				
All 250 frames	39.2	263.0	40.5	316.7
Best 150 frames	15.2	54.6	15.8	65.4

### 3.2 Registration and stitching

We registered PS-OCT data centered on seven retinal positions to one large field set using intensity projection images and a fundus photo as reference. Even though the resulting large field intensity projection image looks very similar to the fundus photo we cannot use the Root-Mean-Square (RMS) error between image pixel values to quantify the performance of our algorithm since we are dealing with multimodal images. Instead we chose a method similar to [40] for grading the accuracy of our registration.

**Fig. 11.**
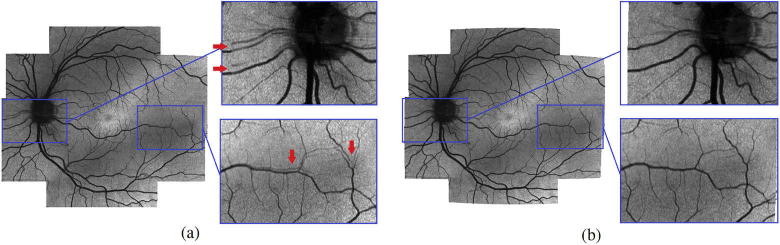
Stitched image of a healthy subject without (a) and with pyramid partitioning (b). Zoomed in regions on the right show the effect of using pyramid partitioning on some retinal vessels. Red arrows indicate areas where vessels can be clearly seen multiple times

On a set of 5 healthy and 5 glaucoma subjects a grader selected 15 points of interest (mainly retinal vessel bifurcations) on each fundus photo. These points were displayed to 3 graders who then had to find the corresponding positions in the stitched intensity projection image. The accuracy of our algorithm is thus given by

(21) $$RMS_{e}=\sqrt{\frac{1}{n}\frac{1}{G}\sum_{i=1}^{n}\sum_{g=1}^{G}{\left||p_{ig}|\right|}^{2}},$$

with n being the number of selected feature points, G the number of graders and ${\left||p_{ig}|\right|}^{2}$ the absolute distance between the $i^{th}$ feature point pair from the $g^{th}$ grader for one image. Using this method we observed a $RMS_{e}$ of the feature point pairs in all 5 healthy subjects of 5.9 Pixel, corresponding to 46.0 μm with a standard deviation of 3.9 Pixel (31.2 μm). For the 5 glaucoma subjects a $RMS_{e}$ of 5.4 Pixel, corresponding to 42.1 μm with a standard deviation of 4.6 Pixel (36.0 μm) was observed. The $RMS_{e}$ of both the healthy and glaucoma set was very similar - suggesting that the registration works similar on both groups. Over all images of both healthy and glaucoma the observed $RMS_{e}$ was 5.6 Pixel (44.1 μm) with a standard deviation of 4.3 Pixel (33.6 μm). Additionally we want to assess the benefit of using our pyramid-based approach in contrast of doing registration without this partitioning. For this, a grader took the set of points acquired in the previous step for the fundus photo and marked their respective locations in the intensity projection images of the individual volumes. Considering that we acquire multiple scans per retinal location, as well as the overlap in between those, a single point marked in the fundus photo can have corresponding points in multiple intensity projection images - typically between 3 and 12. These coordinates are then transformed using the same methods we used to get our large FOV intensity projection images - once including the transformation based on the pyramid partitioning results - and once without. The results thereof are shown in Table 2. The first row gives an impression of how well the transformations between individual scans of a subject coincide. Ideally all corresponding points of a given point in the fundus photo should be on exactly the same spot after transformation - so the standard deviation of those points can indicate how well they coincide with each other. The second and third row give the average distance and standard deviation respectively between the points in the fundus photo and the mean position of the corresponding set of points from the intensity projection images. Figure 11 shows a stitched image of a healthy subject with and without using the pyramid-based approach. It can be seen that both methods yield good stitching results overall, which is reflected in the numerical results given in Table 2. Without using the pyramid partitioning, however, many regions - especially in the periphery - show the retinal vessels twice, because the distortions are not compensated sufficiently, as showcased in the zoomed in regions on the right.

**Table 2.** Comparison of registration performance with and without pyramid partitioning

	without pyramid partitioning		with pyramid partitioning	

	Pixel	μm	Pixel	μm
avg std per point	1.77	13.85	0.89	6.98
avg distance	9.49	74.14	5.29	41.35
avg distance std	2.61	20.40	1.66	12.96

**Fig. 12.**
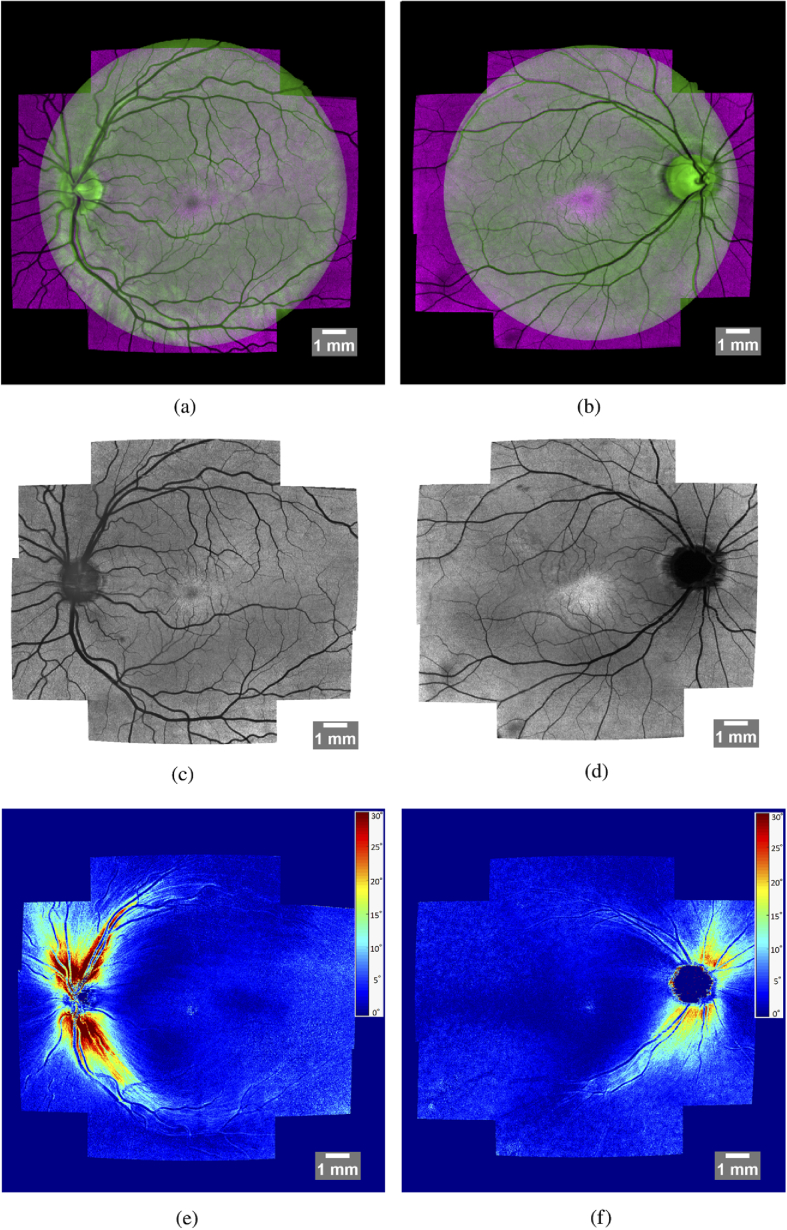
Stitched images of a healthy (left column) and an early glaucoma subject (right column). Overlay of stitched intensity projection image and fundus image (a,b), stitched projection of Intensity (c,d), stitched retardation projection (colorscale in degreeof retardation and windowed between $0^{\circ }$ and $30^{\circ }$) (e,f).

**Fig. 13.**
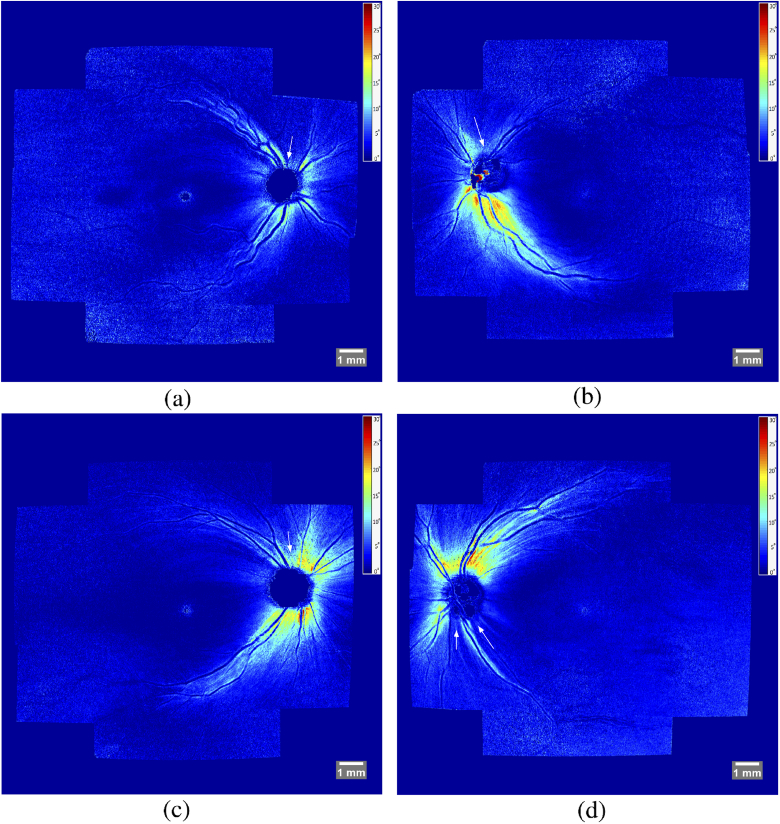
Stitched retardation projection images (colorscale in degree of retardation and windowed between $0^{\circ }$ and $30^{\circ }$) of 4 glaucoma subjects. Bundle defects are indicated with white arrows. Retinal areas associated to these bundle defects will have deficits or even losses in visual perception.

Fig. 12 shows representative results of our complete method for a healthy (left column) and glaucoma (right column) subject. The first row contains a color-coded difference image of a fundus photo (green channel) and the stitched intensity en-face projection image (magenta channel). It can be seen, that these two images fit together very well and only small differences - mostly in the periphery - can be detected. The second row only shows the intensity en-face projection, allowing a more detailed visual inspection of the stitching quality. The third row features a retardation en-face projection which was calculated based on intensity weighted Stokes vector averaging in depth (at the inner/outer segment junction) as was presented in detail in [15]. These retardation en-face images were calculated for each volume and then stitched together to form a large FOV retardation projection image. The technique for stitching the retardation en-face images is the same as was used for stitching intensity projection images. The transformation matrices required for stitching, however, are not recalculated but instead the ones calculated based on the intensity projection images are reused. In these retardation images, the nerve fiber bundles can be clearly visualized, as these are birefringent structures. The loss of nerve fibers in the superior region of the optic disc in the glaucoma patient compared to the healthy case is evident. Figure 13 shows stitched retardation en-face images of 4 additional glaucoma subjects that clearly show defects on the nerve fibers as a result of the progression of the disease. Figure 13(a) shows a case with overall reduced retardation indicating a rather late stage of the disease. Figures 13(b)-d show cases with bundle defects (indicated with a white arrow). Retinal areas associated to these bundle defects will have deficits or even losses in visual perception.

## 4. Discussion

We presented a new method for creating large field projection images of PS-OCT data. The method consists of two key phases - an acquisition phase where volumes are acquired at different retinal positions under the use of a retinal tracker and a stitching phase for estimating the offset and optical distortions of the individual volumes to later seamlessly combine them into one large field map of the image data.

The retinal tracker builds upon our previous work in the field [15]. In terms of tracking accuracy in the fovea region, the new method yields the same performance which is expected as the LSLO imaging speed and transverse resolution of the system are identical. The new algorithm improves the tracking performance when imaging is performed in the periphery of the retina, specifically temporal to the fovea, where retinal features (such as vessel crossings) are rare and typically show low contrast. While the old algorithm frequently failed in these regions, the presented improvements yield a more robust tracking performance. This improvement results from the use of the entire image (equivalent to more information) instead of a small template. One additional aspect is a faster tracking start, as in the previous method the template had to be manually selected by the operator (frequently different locations had to be tested in order to achieve robust tracking) while in the new method the operator just needs to enable tracking. In addition, the re-scan option, in the case of blinks or large eye motion, was implemented which increased the number of usable data sets although this comes at the cost of a slightly increased measurement time. Recently, an interesting concept for re-scanning that is based on OCT-A evaluation has been introduced [41]. As this method is realized without retinal tracking, residual motion artefacts remain which deteriorates quantitative image information and complicates image stitching as proposed in our work. In addition, an OCT-A scanning pattern (i.e. repetitive imaging of the same retinal location) is required which limits the applicability of the method to OCT-A data recording.

Our tracking software utilizes images from the LSLO camera to estimate the retinal movement in real time. One limitation here lies in the LSLO frame rate of 60 Hz and the processing time of 4.5 ms which can accumulate to a delay time between motion and correction of 20 ms. Compared to our previous work [23] we could reduce the processing time by a factor of 2 through the implementation of GPU processing. This further improves the correction efficiency provided by the tracker. Although, this speed is not sufficient to correct for rapid eye motions such as saccades, the tracker is able to correct for typical motion associated with fixation over a couple of seconds including the slow drift as can be seen in Tab. 1. Additionally, the implemented re-take function increases the number of recorded data volumes that show negligible motion artefacts and that can be used for further processing.

Compared to many other systems like the Cirrus HD-OCT tracking system or other custom retinal trackers featuring SLO refresh rates of 20 Hz-30 Hz [42], the tracking performance is better mainly because of the higher LSLO imaging speed and the fast generation of the tracking signal that could be achieved through the implementation of GPU based computation. For a given LSLO or SLO exposuer time, the tracking speed can be increased by splitting the image into multiple narrow stripes - thus reducing the processing time per frame and increasing the update rate of the tracking signal [43]. This approach can in principle be implemented in our setup but it comes at the cost of a reduction of the amount of features that are available in each frame. Thus, potentially the accuracy and robustness of the retinal tracker will be reduced, specifically in peripheral regions where fewer distinct vessel features are present.

It should be noted that even with retinal tracking and the retake function residual artefacts can sometimes still be observed. Figure 10(b) for example shows the compensated volume at a retinal location - yet some small vessel discontinuities can still be seen (cf. the large vessel on the bottom left side of the image). These may be caused by a slight variation of the fixation location of the subject after the blink which results, because of the tracking, in a different beam path that the OCT beam takes through the lens system. Ideally, a B-Scan is obtained by symmetrically scanning of the beam around optical axis of the lens system. However, in the case of a larger beam offset because of varying fixation location and tracking, the lenses are then traversed off-axis which results in a corresponding deterioration of the field of view at locations where the beam traverses the lenses far away from the lens center. This introduces a slight asymmetric skewing or stretching of the B-Scan and the resulting artefacts as can be observed in Fig. 10(b). Even in the case of no blinking, larger deviations from the original fixation location will result in deteriorations of the final pseudo fundus image of the OCT data set. However, these are compensated by the following registration step using the fundus image as reference.

One remaining limitation of the tracker software lies in the type of movement it can compensate. As it is implemented now, the phase correlation approach only accounts for translation ((x,y) movement), not however for rotation. It would be relatively easy to adapt the tracker accordingly by incorporating an additional log-polar transformation in the processing as described in [44]. We deliberately decided against this, mainly because of the sustained processing time. We measured the processing time of all necessary functions and compared that to the timing of the 60 Hz LSLO refresh rate. This yielded an "available processing time" window of about 8ms to 12ms which is completely sufficient for our 4-5ms tracker software. Including the rotation detection in the tracker software, as well as the compensation for the detected rotation in the scanner head trajectory, however, would increase the required processing time and system complexity significantly. Thus, and because none of the subject measurements indicated any noticeable rotation artefacts during acquisition, we chose to not compensate for it.

It should be noted that in this study our retinal layer segmentation algorithm was tested and implemented using healthy and glaucoma subjects. In both cases the retinal layer structures are usually well defined and only differ in thickness (mainly in the RNFL) between healthy and glaucoma subjects. For the segmentation of the RPE we used DOPU images generated from PS-OCT data, where this layer can be seen with high contrast [36]. As we have shown in previous work, this approach yields good performance in other pathologies as well such as geographic atrophy, choroidal neovascularisation or drusen [45–47]. This implies that the proposed methods can be directly translated to these other pathologies.

The stitching algorithm utilizes the OCT intensity projection images as well as a retinal fundus image as reference. Work has already been done for estimating offsets and distortions of multiple images without using a larger image as reference as presented in [48,49], but we felt that the additional information of the fundus photo would prove beneficial with regard to overall robustness. As can be seen in Fig. 12 there aren’t any large discontinuities or stitching seams present in the final images. Two types of artefacts can, however, still be observed. One of them are the line artefacts at and near the optic nerve head. They are a direct result of the segmentation algorithm failing right at the optic nerve head. This causes the averaging window, used for calculating en-face projection images from the RPE, to fall out of place. Consequently, such small artefacts are generated. The other artefact that can be noticed is that some small vessels - especially in the foveal region - are blurred or even doubled. As outlined in Fig. 1, this region contains the most overlapping scans. Currently, individual projection images are registered to the fundus image one after another, without any dependencies in between the images. Thus tiny variations in the individual transformation matrices can quickly lead to such an effect of slightly doubled vessels. This could be improved by calculating transformation matrices globally, as the robustness and accuracy of the algorithm might improve if the estimated offsets of the individual projections are also weighted against one another before calculating the transformation matrix. Another point to consider is the sensitivity of the pyramid-based approach with regard to registration errors carrying over recursively. This is typically not a problem since the region limitation enforces the result to be within at least a reasonable distance to the true offset, it could however cause problems if the very first registration of the entire projection image was affected. In our data this rarely happened and was observed only in very low quality images, but it could prove more problematic with different types of images. In our case we are able to use the prior knowledge of which of the seven retinal locations that scan was recorded at in order to limit the search region based on this estimate.

Finally, we want to point out that the methods introduced here can be used to generate large field of view en-face maps from 3D OCT data of any kind such as intensity data from various layers (RNFL, photoreceptor layer, RPE), polarization sensitive data (DOPU, retardation, axis orientation), or OCTA (superficial plexus, deep plexus, choriocapillaris, choroid).

## 5. Conclusion

In this work, we presented a novel approach for generating large field of view projection images based on volumetric PS-OCT data. For this we acquired PS-OCT volumes at different retinal locations whilst using our custom retinal trackerto minimize motion artefact on the data. These volumes were later stitched together using our custom pyramid-based stitching algorithm and blended to generate large field projections withgreatly reduced motion or stitching artefacts. We later showed that this technique is not limited to generating large field intensity projections, but can also be applied to generate large FOV retinal maps of other data (eg. polarization measurements). The technique can however also be used for generating large FOV retinal maps ofany scanning-based OCT method - especially where one needs a high spatial resolution such as PS-OCT or OCTA.
